# A simpler method for total scalp irradiation: the multijaw‐size concave arc technique

**DOI:** 10.1120/jacmp.v15i4.4786

**Published:** 2014-07-08

**Authors:** Minoru Inoue, Masahiro Konno, Hirofumi Ogawa, Hideyuki Harada, Hirofumi Asakura, Hiroshi Fuji, Shigeyuki Murayama, Tetsuo Nishimura

**Affiliations:** ^1^ Division of Radiation Oncology Shizuoka Cancer Center Hospital Shizuoka Japan; ^2^ Division of Proton Therapy Shizuoka Cancer Center Hospital Shizuoka Japan

**Keywords:** scalp irradiation, conformal arc therapy, cutaneous malignancies, angiosarcoma

## Abstract

The lateral electron‐photon technique (LEPT) and intensity‐modulated radiation therapy (IMRT) are commonly used for total scalp irradiation. However, the treatment planning and irradiation are laborious and time‐consuming. We herein present the multijaw‐size concave arc technique (MCAT) as a total scalp irradiation method that overcomes these problems. CT datasets for eight patients previously treated for angiosarcoma of the scalp were replanned using MCAT, LEPT, and IMRT. The MCAT was designed with a dynamic conformal arc for the total scalp, with a multileaf collimator to shield the brain. Two additional conformal arcs with a decreased upper‐jaw position of the first dynamic conformal arc were used to reduce the cranial hotspots. The prescribed dose was 40 Gy (2 Gy/fraction) to 95% of the planning target volume (PTV, defined as the total scalp plus a 4 mm margin). MCAT was compared with LEPT and IMRT with respect to the PTV dose homogeneity (D5%–95%), underdosage (V < 90%), overdosage (V > 110%), doses to the brain, and the delivery time and monitor units (MUs) for single irradiation. We were able to formulate treatment plans for all three techniques that could deliver the prescription dose in all patients. MCAT was significantly superior to LEPT with respect to PTV dose homogeneity, overdosage, and underdosage, although MCAT was inferior to IMRT with respect to dose homogeneity and overdosage. The mean brain dose and high‐dosage volume of all three techniques were low, but IMRT provided larger volume to the brain than did the other two techniques in the low dosage region. In MCAT, the mean delivery time could be reduced by approximately half or more, and the mean MUs could be reduced by at least 100 compared to the other two techniques. MCAT can achieve total scalp irradiation with substantially fewer MUs and a shorter delivery time than LEPT and IMRT.

PACS number: 87.55.D‐

## I. INTRODUCTION

Cutaneous malignancies of the scalp, such as angiosarcoma, often have poorly defined borders and multifocal spread, and may sometimes invade the skull and forehead. These extensive scalp lesions present a challenge in radiation therapy because of the convex‐shaped target and medially located critical organs (e.g., the brain). To identify the best treatment option, various planning techniques have been developed and tested.

Several electron beam techniques have traditionally been used for total scalp irradiation. The main advantages of electron beams are their high surface dose and rapid dose falloff, offering the potential to decrease the radiation dose to underlying brain tissues. However, matching of adjacent electron fields causes an inhomogeneous dose distribution in the junction area. Many techniques have been reported to address this issue. Able et al.^(1)^ and Mellenberg and Schoeppel^(2)^ used several matching fields and shifted the gap during the course of treatment. Sagar and Pujara^(3)^ and Walker et al.^(4)^ used several overlapping fields to deliver a homogeneous dose at field junctions.

Photon beam approaches have been used to improve the complexities associated with electron beam techniques. Akazawa^(5)^ developed the lateral electron‐photon technique (LEPT) in which the main portions of the lateral scalp are irradiated with one of the two lateral electron fields, and the remainder of the scalp is irradiated with parallel‐opposed photon fields. Subsequently, Tung et al.^(6)^ improved dose uniformity by using overlapped photon and electron fields.

Intensity‐modulated radiation therapy (IMRT) is suitable for the complex target shape in total scalp irradiation. Its effectiveness has been reported in some treatment planning studies. Two studies showed that tomotherapy had a more homogeneous dose distribution to the target compared with LEPT.^7^, ^8^ Bedford et al.^(9)^ compared step‐and‐shoot IMRT to static‐electron and arcing‐electron techniques, and concluded that IMRT provided superior target coverage of the treatment volume, but that doses to the brain and eyes were higher with IMRT. Chan et al.^(10)^ combined static electron fields with photon IMRT, improving target homogeneity, compared with the electron‐only approach, and reducing normal tissue doses compared with photon IMRT alone. Wojcicka et al.^(11)^ noted that step‐and‐shoot IMRT displayed a more homogeneous dose distribution to the target than LEPT. Successful clinical implementation of these techniques has been reported.^9^, ^10^, ^11^, ^12^, ^13^, ^14^

Because the three techniques mentioned above involve a combination of diverse irradiation apertures, treatment planning and irradiation are time‐consuming, imposing a substantial burden on relevant staff members. Furthermore, the monitor units (MUs) for irradiation and the burden on the linear accelerator are high. High‐dose‐rate (HDR) brachytherapy, using a surface mold technique, has been reported as a total scalp irradiation technique for nonexternal therapy since 1999.^15^, ^16^, ^17^ In 2012, a case study reported by Kelly et al.^(18)^ showed that RapidArc achieved total dural irradiation, akin to total scalp irradiation, with lower monitor unit requirements and shorter delivery time compared with static‐field IMRT. However, these techniques require specialized equipment and tools; therefore, only a limited number of institutions can perform these techniques. As a simpler total scalp irradiation technique with photon beams, Kinard et al.^(19)^ applied four 90° photon arcs around the head with central blocks for critical midline structures, but the resulting dose distribution within the scalp seemed inadequate and more heterogeneous. This was partly because multileaf collimators (MLCs) were not available in those days. Nevertheless, arc radiation therapy would naturally suit a spherical target and make the process simpler. As another simpler technique using electron beams, Yaparpalvi et al.^(20)^ used only two or three concentric fields with a single setup, and achieved improved dosimetry at best comparable to that of LEPT and rapid treatment delivery. Note that they used higher beam energies for the fields farther from the center to provide adequate dose homogeneity in the spherical target. By combining aspects of these two techniques, we devised a new total scalp irradiation technique (herein referred to as the “multijaw‐size concave arc technique” (MCAT)) that does not require substantial time to plan or irradiate. This report describes the details of MCAT, and compares it with LEPT and IMRT with respect to the doses to the target and risk organs, delivery time, and MUs.

## II. MATERIALS AND METHODS

### A. Materials and target definition

CT datasets for eight patients who were previously treated with photon or electron beam fields for angiosarcoma of the scalp were selected, and MCAT, LEPT, and IMRT were replanned in the present study. For all three techniques, planning was conducted using Pinnacle^3^ (ver. 9.0; ADAC, Milpitas, CA); the algorithm used for dose calculation was collapsed cone convolution superposition, and the dose calculation grid was set to 2.0 mm. The gross tumor volume (GTV) was defined as the primary tumor. The clinical target volume (CTV) was defined as the GTV and the scalp, which defined as a 3 mm thick ring‐shaped volume from the scalp surface. The caudal level of the CTV was defined as 10 mm above the upper edge of the orbit. When patient setup was performed with head anteflexion, this level was considered to be sufficient to include the occipital scalp, while maintaining an acceptable distance from the eyes. The planning target volume (PTV) was the CTV plus a uniform 4 mm margin, although the caudal level (10 mm above the supraorbit) was not expanded, so as to prevent the PTV from getting close to the eyes. The prescribed dose was 40 Gy (2 Gy/fraction) to 95% of the PTV (D95%), designed to control microscopic disease (so that focused boost radiation could follow it). A Varian Clinac 23EX linear accelerator (Varian Medical Systems, Palo Alto, CA) with a minimal 5 mm MLC (Varian Millennium) was used for all patients in the present comparison study.

### B. Treatment planning

#### B.1 MCAT planning

MCAT plans consisted of up to nine dynamic conformal arcs with a 6 MV photon beam, and these arcs were generated using the following steps.

**Figure 1:**
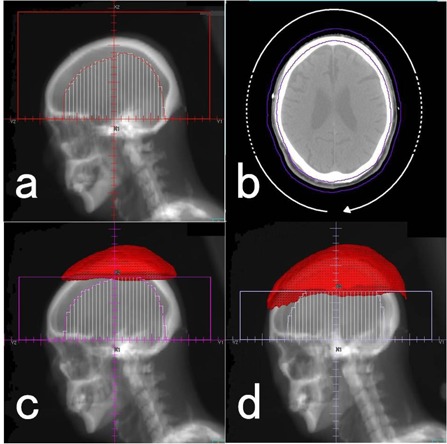
Beam's eye views for MCAT and the path of the gantry: a), c), and d) Beam's eye views for steps 1, 2, and 3, respectively; the 2.0 Gy isodose area is shown in red. The path of the gantry (b) (white circular arrow). The range of the dotted curve can be omitted from the path in the case of anteroposterior hotspots. The PTV is shown as a violet contour. The bolus is not displayed to allow easy identification of the PTV. PTV=planning target volume.

1) The isocenter was centered in the transversal plane of the supraorbit. The MLC aperture for the first arc was shaped to block the structure that contracted the body surface by 3.0 cm (Fig. 1(a)). The gantry was rotated from 181° to 179° counterclockwise during the arc treatment, and this arc was set to deliver approximately 250 MUs. To ensure an adequate skin dose, a 10 mm thick tissue‐equivalent bolus was used. When an isodose line greater than 2.5 Gy was detected around the anterior or posterior edge of the head, this arc was divided into three parts to evacuate the hotspots (e.g., from 181° to 260°, from 280° to 80°, and from 100° to 179°; see also Fig. 1(b)).
2) The second arc was copied from the first arc, and the upper jaw (X2 jaw) was moved to the bottom of the 2.0 Gy isodose line given by the first arc (Fig. 1(c)). This arc was set to deliver approximately 90 MUs. The rotation angle of the gantry was adjusted as noted above.
3) The third arc was copied from the second arc, and the X2 jaw was moved to the bottom of the 2.0 Gy isodose line given by the first and second arcs (Fig. 1(d)). This arc was set to deliver approximately 100 MUs. The rotation angle of the gantry was adjusted as noted above. Importantly, throughout the steps, the dose rate for each arc should be kept >0.3 MU per degree (the minimum dose rate of our linac for dynamic arc irradiation) and MLC leaf motion between adjacent control points should be kept small and smooth.

Two of the eight MCAT plans were verified with films (Ready Pack Kodak EDR‐2; Carestream Health, Rochester, NY). To acquire the coronal dose distribution, the film was positioned at a depth of 10 cm in five 40×40×5 cm pieces (total thickness, 25 cm) Solid Water phantoms; to acquire the axial dose distribution, the film was positioned 1 cm below the cranial edge of the third arc in a 30 cm diameter cylindrical phantom. An EPSON ES‐10000G scanner (EPSON Offirio ES‐10000G; US Epson, Long Beach, CA) was used to scan the films. Analysis was performed using DD‐System ver. 9 (R‐Tech Ueno Ltd., Tokyo, Japan).

#### B.2 IMRT planning

Inversely planned IMRT was performed with a step‐and‐shoot technique. Nine coplanar beams with gantry angles of 20°, 60°, 100°, 140°, 180°, 220°, 260°, 300°, and 340° were used. A 10 mm thick tissue‐equivalent bolus was used. In addition to the common structures, the 10 mm width of the ring‐shaped region of interest (ROI) was set at 10 mm inside the PTV (defined as inner ring 1), the 5 mm width of the ring‐shaped ROI was set at 5 mm inside inner ring 1 (defined as inner ring 2), and then inner rings 1 and 2 were subtracted from the brain (defined as the medial brain).

Our typical inverse planning objectives were as follows: dose received by 95% of the PTV>41 Gy(relative weight(w)=95), uniform dose of 42 Gy to the PTV(w=90), dose received by 5% of the PTV<44 Gy(w=85), minimum irradiated dose ($D_{\text{min}}$) of the PTV>40 Gy(w=100), maximum irradiated dose ($D_{\text{max}}$) of the medial brain <30 Gy(w=40), dose received by 10% of the medial brain <20 Gy(w=30), dose received by 30% of the medial brain <10 Gy(w=10), dose received by 80% of the medial brain <5 Gy(w=5), $D_{\text{max}}$ of inner ring 1<39 Gy(w=50), $D_{\text{max}}$ of inner ring 2<35 Gy(w=45), $D_{\text{max}}$ of the right eye <4 Gy(w=10), and $D_{\text{max}}$ of the lefteye <4 Gy(w=10).

#### B.3 LEPT planning

LEPT plans comprised lateral photon and electron beams, as described previously.^(6)^ The inner field was a 9 MeV electron beam. The outer semicircular field was a 6 MV photon beam that overlapped with the electron beams by 3 mm at the photon‐electron abutment region. The treatment plan included a 1 cm shift in the junction after ten treatments.

### C. Comparisons

Quantitative evaluation of plans was performed using dose‐volume histograms (DVHs). For the PTV, the dose received by 95% of the PTV (D95%) and the dose received by 5% of the PTV (D5%) were calculated. The difference between the D5% and D95%, D5%‐D95%, was used as a parameter indicating the homogeneity of the dose distribution. The volumes receiving less than 90% of the prescribed dose (V<90%) and more than 110% of the prescribed dose (V>110%) were used as parameters indicating underdosages and overdosages, respectively. For risk organs, the mean brain dose, percentages of the brain volume exceeding 10 Gy and 40 Gy (V10Gy and V40Gy, respectively), and maximum dose ($D_{\text{max}}$) to the eyes and lenses were calculated.

The delivery time, which was defined as the time from “beam‐on” at the first irradiation aperture to the completion of irradiation at the last irradiation aperture, was measured for each irradiation technique. In LEPT, the time required to attach the electron beam applicator was included in the measurement. The MU level for a single irradiation was used to evaluate the burden on the linear accelerator. The MUs calculated for the treatment planning system were compared among the three techniques.

Results were analyzed using a two‐sided, paired *t*‐test, and values were considered statistically significant if p=<0.05. All analyses were performed with the ‘R’ software (ver. 2.13.1; http://www.R-project.org).

## III. RESULTS

**Figure 2:**
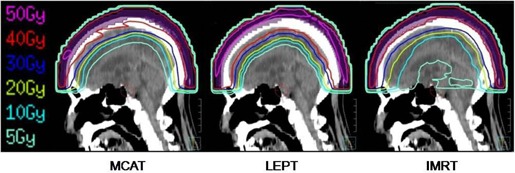
Typical isodose distribution for the three techniques. The PTV is shown as a violet contour. The bolus is not displayed to allow easy identification of the isodose lines. PTV=planning target volume; MCAT=multijaw‐size concave arc technique; LEPT=lateral electron‐photon technique; IMRT=intensity‐modulated radiation therapy.

**Figure 3:**
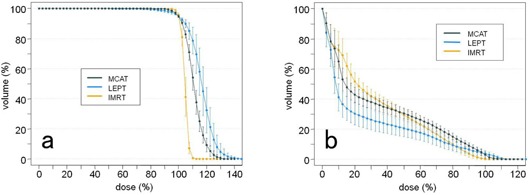
Mean DVHs of the PTV (a) and brain (b) for the three techniques. The error bar indicates one standard deviation. DVHs=dose‐volume histograms; PTV=planning target volume; MCAT=multijaw‐size concave arc technique; LEPT=lateral electron‐photon technique; IMRT=intensity‐modulated radiation therapy.

We were able to formulate treatment plans for all three techniques that could deliver the prescription dose in all patients. The details of each MCAT plan are listed in Table 1. Typical isodose distributions and mean DVHs regarding the PTV and brain are shown in Figs. 2, 3(a), and 3(b). Mean values of the DVH parameters (i.e., for the PTV: D5%–D95%, V<90%, and V>110%; for the brain: mean brain dose, V10Gy, and V40Gy; for eyes and lenses: D_max_) are shown in Table 2. MCAT was significantly superior to LEPT with respect to PTV dose homogeneity, overdosage, and underdosage (i.e., D5%–D95%, V<90%, and V>110%, respectively). However, MCAT was inferior to IMRT with respect to dose homogeneity and overdosage. Regarding the brain, the mean V10Gy value was significantly higher with MCAT than with LEPT, but was lower with MCAT than with IMRT. The mean V40Gy value showed no significant difference between MCAT and LEPT, but showed a slightly, but significant, difference between MCAT and IMRT. The average $D_{\text{max}}$ values of eyes and lenses for both techniques were negligibly small.

**Figure 4:**
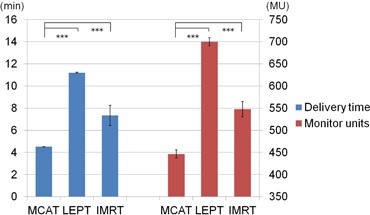
Mean delivery time (left) and monitor units (right). The error bar indicates one standard deviation. MCAT=multijaw‐size concave arc technique; LEPT=lateral electron‐photon technique; IMRT=intensity‐modulated radiation therapy ***p<0.001.

Figure 4 shows the mean delivery time and MUs of each irradiation technique. MCAT had a significantly shorter delivery time and lower MUs than the other two techniques.

The measured relative distributions agreed with those planned to ± 5%.

**Table 1:** Gantry angles and prescribed monitor units for each conformal arc in the MCAT plans

	*Arc(s) Generated in Step 1*		*Arc(s) Generated in Step 2*		*Arc(s) Generated in Step 3*	
*Patient*	*Gantry Angle*	*MU*	*Gantry Angle*	*MU*	*Gantry Angle*	*MU*
1	181°–179°	250	181°–255°	23	181°–255°	25
			285°–75°	45	285°–75°	50
			105°–179°	23	105°–179°	25
2	181°–179°	260	181°–255°	24	181°–255°	23
			285°–75°	48	285°–75°	50
			105°–179°	28	105°–179°	32
3	181°–179°	250	181°–255°	23	181°–255°	29
			285°–75°	45	285°–75°	44
			105°–179°	23	105°–179°	37
4	181°–179°	250	181°–255°	23	181°–255°	29
			285°–75°	45	285°–75°	50
			105°–179°	23	105°–179°	32
5	181°–179°	250	181°–250°	27	181°–250°	29
			300°–60°	36	300°–60°	38
			110°–179°	27	110°–179°	29
6	181°–179°	250	181°–255°	23	181°–245°	25
			285°–75°	45	295°–65°	50
			105°–179°	23	115°–179°	25
7	181°‐179°	250	181°–250°	24	181°‐250°	25
			300°–60°	48	300°–60°	50
			110°°179°	24	110°–179°	25
8	181°–265°	63	181°–255°	23	181°–245°	25
	275°–85°	125	285°–75°	45	295°–65°	50
	95°–179°	63	105°–179°	23	115°–179°	25

MCAT=multijaw−size concave arc technique; MU=monitor unit.

**Table 2:** Summary of dosimetric results for MCAT, LEPT, and IMRT plans

*Volume of Interest*	*Description*	*MCAT mean (range)*	*LEPT mean (range)*	*p* ^a^	*IMRT mean (range)*	*p* ^b^
PTV	D5%–95% (%)	23.8	31.3	0.01	8.0	<0.001
		(20.4–28.2)	(23.0–39.9)		(5.9–9.0)	
	V&lt;90% (%)	0.2	1.6	0.004	0.0	0.03
		(0.0–0.6)	(0.0–2.7)		(0.0–0.3)	
	V<110% (%)	54.9	81.5	<0.001	0.7	<0.001
		(42.0–64.3)	(63.6–95.0)		0.0–1.6)	
Brain	Mean dose (Gy)	13.6	10.0	<0.001	13.8	0.80
		(11.2–15.3)	(6.1–12.1)		(10.5–15.2)	
	V10Gy (%)	41.2	28.2	<0.001	46.0	0.002
		(33.7–48.1)	(17.5–37.8)		(39.0–53.0)	
	V40Gy (%)	3.0	3.9	0.31	0.0	0.004
		(0.6–7.0)	(0.2–7.4)		(0.0–0.2)	
Eye						
Right	Max dose (Gy)	3.1	3.5	0.33	2.9	0.04
		(2.1–4.6)	(2.1–5.7)		(1.9–4.5)	
Left	Max dose (Gy)	3.1	3.1	0.98	2.9	0.04
		(2.2–4.6)	(2.0–5.6)		(2.0–4.8)	
Lens						
Right	Max dose (Gy)	1.4	1.2	0.01	1.3	0.005
		(1.2–1.5)	(1.1–1.3)		(1.2–1.4)	
Left	Max dose (Gy)	1.3	1.1	0.11	1.2	
		(1.0–1.5)	(1.0–1.3)		(1.0–1.5)	0.05

a P‐values reported here were obtained using paired t‐tests comparing MCAT with LEPT.

b P‐values reported here were obtained using paired t‐tests comparing MCAT with IMRT.

PTV=planning target volume; MCAT=multijaw‐size concave arc technique;

LEPT=lateral electron‐photon technique; IMRT=intensity‐modulated radiation therapy.

## IV. DISCUSSION

In this report, we have described the mechanics of MCAT planning. The simplicity of this technique is that it can be applied by rotating only three types of fields with different upper‐jaw positions and requires only three gantry rotations. According to our results, all MCAT plans need to divide a 360° arc into three shorter arc segments to reduce anteroposterior hotspots. It might be iterative to tweak the gantry angle to individualize the treatment plan, but as shown in Table 1, the pattern of the arc dividing is similar in almost all cases. In our experience, the time required for MCAT planning is a few hours; therefore, MCAT planning is simpler than that for IMRT, which requires the iterative application of the dose constraints, or that of LEPT, which requires a combination of X‐rays and electron beams.

Furthermore, our results show that the delivery time and MUs for single irradiation in MCAT could be reduced substantially, compared with the other two techniques. The mean delivery time of MCAT was reduced by approximately half or more, and the MUs by more than 100, compared with the other two techniques. These substantial reductions will greatly decrease the burden on radiological technologists and the linear accelerator.

Regarding the PTV dosage, no major differences were noted in underdosage among the three techniques, indicating that the dose to the PTV was sufficient. Regarding dose homogeneity and overdosage, MCAT was substantially better than LEPT, but significantly inferior to IMRT. A possible reason for this is that the MU level was determined to prevent lowering of the PTV dosage, resulting in the generation of small, high‐dosage regions that were difficult to evacuate. Nevertheless, because the skin in these high‐dose regions can tolerate high dosages relatively well, no major problems are likely to occur in actual clinical settings. Accordingly, this will not be a major disadvantage for MCAT.

The mean brain dose and high dosage volume (V40Gy) of all three techniques were low. Furthermore, the differences in these parameters among the three techniques were so small that they may be clinically negligible. Meanwhile, comparison of the low dosage volume (V10Gy) showed a larger difference, compared with the two dosage parameters mentioned above. However, the probable/definitive effect of low dosage exposure by the brain has not been elucidated. Therefore, we are unable to discuss the superiority or inferiority of the three techniques from the perspective of the low dosage volume.

Because no normalization point is commonly available for all irradiation apertures of MCAT, we set the MUs for each irradiation aperture (Table 1). Therefore, it should be confirmed whether there is any difference between the dosage calculated by the treatment planning system and the actual dosage. According to the film measurement, the calculated dose distribution for MCAT is reliable.

## V. CONCLUSIONS

This article describes a simple treatment planning technique for total scalp irradiation — the multijaw‐size concave arc technique (MCAT). MCAT readily provides an adequate dose to the total scalp, while sparing the brain and optical structures. In addition, it demonstrates considerable reduction in delivery time and MUs, compared with LEPT and IMRT. MCAT is thus an acceptable alternative to LEPT or IMRT.

## ACKNOWLEDGMENTS

We are thankful to Dr. Yoshio Kiyohara and Dr. Shusuke Yoshikawa, Division of Dermatology, Shizuoka Cancer Center Hospital, for their clinical support.
